# Burden of rare genetic disorders in India: twenty-two years’ experience of a tertiary centre

**DOI:** 10.1186/s13023-024-03300-z

**Published:** 2024-08-13

**Authors:** Jayesh Sheth, Aadhira Nair, Frenny Sheth, Manali Ajagekar, Tejasvi Dhondekar, Inusha Panigrahi, Ashish Bavdekar, Sheela Nampoothiri, Chaitanya Datar, Ajit Gandhi, Mamta Muranjan, Anupriya Kaur, Manisha Desai, Mehul Mistri, Chitra Patel, Premal Naik, Maulin Shah, Koumudi Godbole, Seema Kapoor, Neerja Gupta, Sunita Bijarnia-Mahay, Sandeep Kadam, Dhaval Solanki, Soham Desai, Anand Iyer, Ketan Patel, Harsh Patel, Raju C. Shah, Shalmi Mehta, Ruchi Shah, Riddhi Bhavsar, Jhanvi Shah, Mili Pandya, Bhagyadhan Patel, Sudhir Shah, Heli Shah, Shalin Shah, Shruti Bajaj, Siddharth Shah, Nilam Thaker, Umesh Kalane, Mahesh Kamate, Vykunta Raju KN, Naresh Tayade, Sujatha Jagadeesan, Deepika Jain, Mitesh Chandarana, Jitendra Singh, Sanjiv Mehta, Beena Suresh, Harsh Sheth

**Affiliations:** 1 FRIGE Institute of Human Genetics, FRIGE House, Ahmedabad, India; 2 Postgraduate Institute of Medical Education and Research, PGIMER, Chandigarh, India; 3 Department of Pediatrics, K.E.M Hospital, Pune, India; 4 Department of Paediatrics, Amrita School of Medicine, Kochi, India; 5 Bharati Hospital and Research Centre, Dhankawadi, Pune India; 6 Unique Hospital, Solapur, India; 7 Department of Pediatrics, KEM Hospital, Parel, Mumbai India; 8 Rainbow Super speciality Hospital, Ahmedabad, India; 9 Ortho Kids Clinic, Ahmedabad, India; 10 Deenanath Mangeshkar Hospital & Research Centre, Pune, India; 11 Division of Genetics & Metabolism Department of Pediatrics, Lok Nayak Hospital and Maulana Azad Medical College, New Delhi, India; 12 Division of Genetics, Department of Pediatrics, All India Institute of Medical Sciences, New Delhi, India; 13 Institute of Medical Genetics and Genomics, Sir Ganga Ram Hospital, New Delhi, India; 14 Nirmal Mantra Children’s Hospital, Bhavnagar, India; 15 Shree Krishna Hospital, Karamsad, Anand India; 16 Neuro Kids Clinics, Ahmedabad, India; 17 Himalaya Arcade, Homeopathy Clinic, Vastrapur, Ahmedabad India; 18 Zydus Hospital & Healthcare Research Pvt Ltd, Ahmedabad, India; 19 Ankur Neonatal Hospital, Ashram Road, Ahmedabad, India; 20 Endokids Clinic, Ahmedabad, India; 21 Brain and Nerve Clinic, Sola, Ahmedabad India; 22 NHL Medical College, Ahmedabad, India; 23 Ansa Clinic, S. G. Highway, Ahmedabad, India; 24 The Purple Gene Clinic, Simplex Khushaangan, SV Road, Malad West, Mumbai, India; 25 RICN Hospital, Ahmedabad, India; 26 Pediatric Nephrologist, Ahmedabad, India; 27 KLES Prabhakar Kore Hospital, Belgaum, India; 28 Department of Pediatric Neurology, Indira Gandhi Institute of Child Health, Bangalore, India; 29 Department of Paediatrics, Dr. Panjabrao Deshmukh Memorial Medical College, Amravati, India; 30 Department of Clinical Genetics & Genetic Counselling, Mediscan Systems, Chennai, India; 31 Shishu Child Development and Early Intervention Centre, Ahmedabad, India; 32 Medisquare Superspeciality Hospital and Research Institute, Ahmedabad, India; 33 Neurology Clinic, Shivranjini Cross Road, Satellite, Ahmedabad India

**Keywords:** Rare diseases, Diagnosis, IEM, Neuromuscular, Neurodevelopmental disorders, Lysosomal storage diseases, Prevalence, Common variant, Founder variant, India

## Abstract

**Background:**

Rare disorders comprise of ~ 7500 different conditions affecting multiple systems. Diagnosis of rare diseases is complex due to dearth of specialized medical professionals, testing labs and limited therapeutic options. There is scarcity of data on the prevalence of rare diseases in different populations. India being home to a large population comprising of 4600 population groups, of which several thousand are endogamous, is likely to have a high burden of rare diseases. The present study provides a retrospective overview of a cohort of patients with rare genetic diseases identified at a tertiary genetic test centre in India.

**Results:**

Overall, 3294 patients with 305 rare diseases were identified in the present study cohort. These were categorized into 14 disease groups based on the major organ/ organ system affected. Highest number of rare diseases (D = 149/305, 48.9%) were identified in the neuromuscular and neurodevelopmental (NMND) group followed by inborn errors of metabolism (IEM) (D = 47/305; 15.4%). Majority patients in the present cohort (*N* = 1992, 61%) were diagnosed under IEM group, of which Gaucher disease constituted maximum cases (*N* = 224, 11.2%). Under the NMND group, Duchenne muscular dystrophy (*N* = 291/885, 32.9%), trinucleotide repeat expansion disorders (*N* = 242/885; 27.3%) and spinal muscular atrophy (*N* = 141/885, 15.9%) were the most common. Majority cases of β-thalassemia (*N* = 120/149, 80.5%) and cystic fibrosis (*N* = 74/75, 98.7%) under the haematological and pulmonary groups were observed, respectively. Founder variants were identified for Tay-Sachs disease and mucopolysaccharidosis IVA diseases. Recurrent variants for Gaucher disease (*GBA*:c.1448T > C), β-thalassemia (*HBB*:c.92.+5G > C), non-syndromic hearing loss (*GJB2*:c.71G > A), albinism (*TYR*:c.832 C > T), congenital adrenal hyperplasia (*CYP21A2*:c.29–13 C > G) and progressive pseudo rheumatoid dysplasia (*CCN6*:c.298T > A) were observed in the present study.

**Conclusion:**

The present retrospective study of rare disease patients diagnosed at a tertiary genetic test centre provides first insight into the distribution of rare genetic diseases across the country. This information will likely aid in drafting future health policies, including newborn screening programs, development of target specific panel for affordable diagnosis of rare diseases and eventually build a platform for devising novel treatment strategies for rare diseases.

**Supplementary Information:**

The online version contains supplementary material available at 10.1186/s13023-024-03300-z.

## Background

Rare diseases affect multiple organ systems and present with a range of phenotypes, majority (~ 50–75%) of them affecting children. Presently, there are ~ 7500 rare diseases for which molecular basis is known that collectively are estimated to affect ~ 300 million people worldwide [1]. Nearly 80% of all rare diseases are estimated to have an underlying genetic aetiology [2].

The diagnosis and treatment of rare genetic diseases is complex due to poor awareness among healthcare providers [3, 4], dearth of testing laboratories and unavailability of therapy for majority of them. Advancements in genomic technologies, especially, emergence of high throughput technologies like DNA microarray analysis and next generation sequencing (NGS) have identified several novel genes which are associated with rare genetic diseases that has led to improvement in diagnostic yields. In addition, international initiatives such as NIH Undiagnosed Diseases Program and Network and The International Rare Diseases Research Consortium have been undertaken [5, 6] in order to delineate aetiology of rare genetic diseases, and accelerate research for discovery of novel genetic conditions and possible therapeutics.

Statistics on the prevalence of rare genetic diseases in different populations is scarce. Walker et al. 2016 in Western Australia did one of the first pioneering attempts to estimate the collective prevalence and burden of rare diseases in a large population. The study observed that ~ 2% of the population of Western Australia is affected by a rare disease [7]. Similarly, a rare disease prevalence of 1.5% was reported in Hong Kong [8]. Interestingly, a study by Hsu et al. 2018 in Taiwan showed that the estimated prevalence of rare diseases increased at an average rate of 19.46% per year [9].

The available epidemiology data for rare diseases is mostly from data by national registries or small cohorts on single diseases or disease groups [1]. A recent study on neuromuscular patient cohort from Lebanon identified 62 rare genetic neuromuscular disorders (NMD). The group reported a high incidence of approximately 1 in 7500 births for spinal muscular atrophy (SMA) in Lebanon [10]. Likewise, a retrospective study carried out in Australia found a combined incidence of lysosomal storage disorders (LSDs) to be approximately 1 per 4,800 live births, with Fabry disease observed as the most common LSD [11].

India has the largest population in the world comprising of 4600 population groups which is stratified into several tribes and castes based on the socio-cultural background and geographical location [12, 13]. The strict inbreeding practices among certain communities has resulted in high prevalence of several genetic rare diseases and associated founder variants [14]. Despite this, limited information is presently available on the prevalence of rare genetic diseases in India due to the lack of a centralized patient registry, diagnostic facility, affordable therapeutic interventions and awareness. The Foundation for Research on Rare Diseases and Disorders has estimated that about 70 million people are affected with rare genetic diseases in India [15]. The Indian Council of Medical Research (ICMR) has recently set up a National Registry for Rare Diseases, which compiles epidemiological data on rare diseases and estimates that there are 4,001 identified rare diseases [16]. This data and the observations by several groups working on rare diseases in India have shown the most prevalent among rare diseases to be haematological disorders, lysosomal storage disorders (LSDs), neuromuscular and neurodegenerative disorders (NMND), primary immunodeficiency diseases (PID) and mitochondrial diseases [17]. For example, Yadav et al. 2022 has shown a high prevalence of β-thalassemia (β-thal) trait in central India, ranging between 1.4 and 3.4% [18]. Likewise, the prevalence of haemophilia A in India is estimated to be 0.9 per 100,000 people [19]. Furthermore, recent studies have estimated the incidence of SMA to be 1 in 10,000 newborns [20] and that of cystic fibrosis (CF) to be between 1 in 40,000 and 1 in 100,000 [21].

Considering the large and heterogeneous population in India, studying the epidemiology of rare genetic disorders is important in order to provide appropriate, timely, cost-effective diagnosis and targeted therapeutic interventions to affected patients. Till date, no single study has assessed the collective prevalence or burden of different rare diseases at a national/ state or a tertiary centre level. The present study provides a 22-year retrospective overview of a cohort of patients with rare genetic diseases identified at a tertiary genetic centre in India. To the best of our knowledge this is a first of its kind systematic study within the Indian population.

## Methods

### Patient cohort detail

This is a retrospective study carried out at FRIGE Institute of Human Genetics, a tertiary referral centre, from 2000 to 2022. Patients with a strong clinical presentation, positive primary investigations and a confirmatory molecular or biochemical test were included in the present study. The institutional ethics committee of FRIGE’s Institute of Human Genetics approved this study. All procedures performed in studies were in accordance with the ethical morals of the 1975 Helsinki declaration.

For all patients, 4–5 millilitres of peripheral blood sample was collected in an EDTA vacutainer after obtaining an informed consent. This was used for isolating genomic DNA using the salting-out method [22]. Genomic DNA was quantified using QIAexpert (Qiagen, Germany) and stored at -20 ֯C until further molecular studies. For patients suspected with inborn errors of metabolism, in addition to the peripheral blood sample, urine and/or plasma samples were also collected. For the purpose of biochemical investigations in patients suspected with LSDs, leukocytes were isolated from whole blood.

**Fig. 1:**
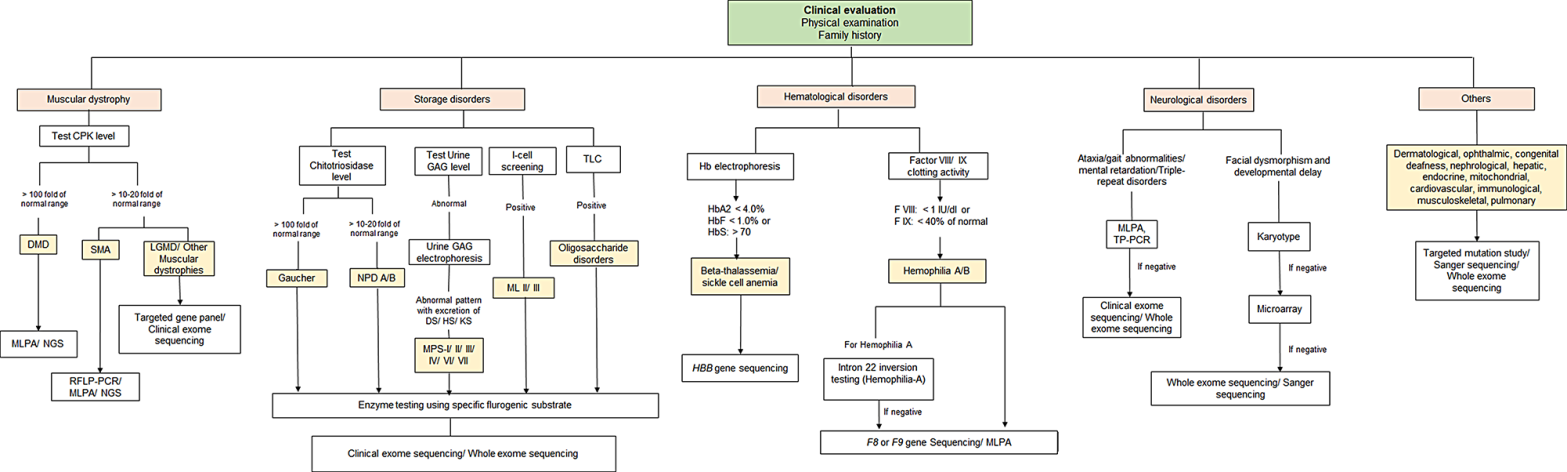
Overview of the diagnostic test pathway used for testing rare genetic diseases. CPK = creatine phosphokinase; DMD = Duchenne muscular dystrophy; SMA; spinal muscular atrophy; LGMD = Limb girdle muscular dystrophy; MLPA = Multiplex ligation probe dependent amplification; NGS = Next generation sequencing; RFLP-PCR = Restriction fragment length polymorphism- polymerase chain reaction; GAG = Glycosaminoglycan; NPD = Niemman-Pick disease; MPS = Mucopolysaccharidosis; ML = Mucolipidosis; TP-PCR = Triplet prime repeat polymerase chain reaction

Briefly, 1 ml of blood sample was added to acid-citrate-dextrose (ACD) solution and incubated for 1 h. The supernatant containing the leukocyte was collected in a separate vial and subjected to centrifugation to settle the pellet. Two rounds of normal saline washes were given to the pellet and stored at -20 ֯C until further testing. The line of investigation strategy utilized for the diagnosis was dictated by the preliminary clinical suspicion or the type of rare genetic disorder suspected by the referring clinician (Fig. 1).

### Muscular dystrophy

Patients with a primary suspicion of muscular dystrophy were first tested for their serum creatine phosphokinase (CPK) levels [23]. Patient samples with elevated CPK levels were then processed for molecular testing of DMD/ Becker muscular dystrophy (BMD). For this, DNA samples were subjected to multiplex PCR to detect hemizygous deletion in the *DMD* gene [24]. Reflex testing by multiplex ligation-dependent probe amplification (MLPA) was performed in cases whereby hemizygous deletion in the *DMD* gene was not observed [25] in accordance with the manufacturer’s protocol of SALSA MLPA probemix P034-A3/P035-A3 DMD/Becker (MRC Holland, Netherlands). Patient samples that remained undiagnosed after carrying out the above tests and those with a clinical suspicion of muscular dystrophies were subjected to NGS based test (Details provided in the following section titled clinical exome sequencing/ whole exome sequencing).

### Motor neuron disease

For patients with a primary suspicion of SMA, their DNA was subjected to Restriction fragment length polymorphism (RFLP)-PCR as per the protocol described previously [26] or MLPA using SALSA MLPA Probe mixes; P021-A2 or P021-B1 (MRC-Holland, Netherlands). DNA of patients suspected with spinal and bulbar muscular atrophy were subjected to Sanger sequencing to assess the trinucleotide CAG repeats in the *AR* gene as described by Spada et al. 1991 [27].

### IEM disorders

Preliminary screening tests were performed for different LSDs based on the clinical differential diagnosis as described in Fig. 1. Plasma chitotriosidase testing was performed using 4-methyl umbelliferyl (4-MU) fluorometric assay [28, 29]. Glycosaminoglycan levels in urine samples were tested (quantitative and qualitative) in MPS suspected patients [30]. I-cell screening and thin layer chromatography was performed from plasma and urine sample, respectively, using previously described methodology [31].

Enzyme assay from leukocytes and/ or plasma of screening positive patients was performed by standard protocol for a given enzyme using 4-MU fluorometric assay or para-nitrocatechol sulfate (PNCS) spectrophotometric synthetic substrate as outlined previously [32, 33].

Molecular genetic study in patients with a positive enzymatic assay result was carried out by Sanger sequencing. The region of interest was amplified using specific primers designed with Primer3 tool (https://bioinfo.ut.ee/primer3-0.4.0/) [34]. Following this, bi-directional Sanger sequencing using ABI SeqStudio platform (Thermo Fisher Scientific, USA) was performed. List of genes for which Sanger sequencing was performed is mentioned in Additional file 1.

For the detection of other IEM disorders like amino acid disorders, organic acidemias and fatty acid oxidation disorders, clinical exome sequencing (CES) study was performed as described in the following section. Patients with IEM disorders identified by tandem mass spectrometry (TMS) or gas chromatography mass spectrometry (GCMS) were excluded from the present study.

### Haematological disorders

A preliminary Hb electrophoresis test was performed in patients with a suspicion and/or family history of β-thalassemia and/or sickle cell anaemia [35]. DNA samples of patients with positive Hb electrophoresis test result were processed for the detection of variants in the *HBB* gene by Sanger sequencing using exon specific primers (Additional file 1).

Factor VIII/ IX clotting activity was assessed in plasma samples of patients suspected with haemophilia. Molecular genetic study was performed for the detection of the common variant in haemophilia A patients: intron 22 inversion of the *F8* gene using the inverse PCR protocol described previously [38]. Sanger sequencing using exon-specific primers was performed for the detection of other variants in the *F8* and *F9* gene (Additional file 1).

### Trinucleotide repeat expansion disorders

For the molecular genetic diagnosis of myotonic dystrophy type I and type II and Fragile-X, triplet repeat primed PCR followed by fragment length analysis was performed as per the protocol described previously [39, 40]. End-point PCR was performed for the diagnosis of Huntington’s disease, Friedreich’s ataxia and spinocerebellar ataxias (1, 2, 3, 6, 7, 12 and 17) using region specific primers [41–44].

### Cystic fibrosis

For the detection of the common variant c.1521_1523del in the *CFTR* gene, ARMS-PCR protocol as described previously was performed in all patients that were referred with a clinical suspicion of cystic fibrosis [45]. If the patient was not detected with the *CFTR*:c.1521_1523del variant, Sanger sequencing using exon-specific primers was performed for the detection of causative variants in the *CFTR* gene (Additional file 1).

### Clinical exome sequencing/ whole exome sequencing

For patients with unusual clinical presentation and/ or a strong clinical suspicion for a rare genetic disease which are mentioned above, clinical exome sequencing (CES) was performed. Whole exome sequencing was performed in cases with overlapping, complex and uncertain phenotypes. CES included exon and splice site regions of > 6670 genes associated with known inherited diseases, captured using a custom capture kit (Additional file 2). Paired-end 150 bp sequencing was carried out on Illumina sequencing platforms (Illumina, USA) in accordance with the manufacturer’s protocols, with an average coverage of the target regions with ~ 100x. For whole exome sequencing (WES), genomic DNA of the proband was subjected to selective capture and sequencing of the protein coding regions that included exons and exon-intron boundaries of genes using either Agilent SureSelect v6 enrichment kit (Agilent, USA) or Twist Human Core Exome kit (Twist Biosciences, USA). The prepared library was subjected to paired-end sequencing with a mean coverage of ~ 100x on the Illumina HiSeq 2500 or NovaSeq 6000 platform (Illumina, USA).

Sequences from CES or WES obtained as FASTQ files were aligned using BWA MEM v0.7.12 [46] to the human reference genome (GRCh37/hg19). SNVs and indels were called using GATK Haplotype caller [47]. Additionally, copy number variants (CNVs) were detected from the data using the ExomeDepth v1.1.10 [48]. Variant annotation, filtration and prioritization was performed based on a given set of Human Phenotype Ontology coded phenotype terms using Exomiser using hiPHIVE prioritisation methodology [49]. Common variants were filtered based on minor allele frequency > 1% in the 1000Genome Phase 3, TopMed and/or gnomAD databases. Only non-synonymous variants in the coding region and canonical splice site variants with a depth of > 20x were used for analysis and clinical correlation. Various in-silico prediction tools such as PolyPhen-2 [50], SIFT [51], MutationTaster2 [52] and CADD [53] were used to predict pathogenicity of non-synonymous and indel variants. Post-gross filtering, variants were prioritised based on the following: (a) known disease causing variant previously reported in databases like ClinVar, and; (b) novel variants in known genes based on the Z-score for missense and pLOF or LOEUF score for loss of function variants available in the gnomAD database. In the case of candidate CNVs, variants were primarily screened for population frequency and known disease associations using publicly available databases like gnomAD, DGV, DECIPHER and OMIM. All the variants identified were classified in accordance with the American College of Medical Genetics-American College of Pathologists (ACMG-AMP) and ClinGen classification system [54, 55].

## Results

### Patient cohort details

A total of 7481 patients were referred for genetic test between the year 2000 and 2022. Of this, 3294 patients, including 2141 males (65%) and 1153 females (35%) were diagnosed with a rare genetic disorder. The age of the patients at the time of diagnosis ranged from 1 day to 74 years. Table 1 provides age-group wise distribution of all patients along with the male to female ratio under each group. State-wise distribution of positive cases based on their referral clinic is presented in Additional file 3. The highest number of diagnosed cases were referred from Gujarat (*N* = 1601; 48.6%), followed by Maharashtra (*N* = 825; 25%), Karnataka (*N* = 226; 6.9%), Kerala (*N* = 164; 4.9%) and Punjab (*N* = 163; 4.9%). A poor representation of only 33 diagnosed cases was from the eastern states of the country- namely West Bengal, Odisha, and Chhattisgarh.

**Table 1:** Stratification of disease groups by age and sex

Disease group	< 1 year	> 1 to 12 years	> 12 to 18 years	> 18 years and above	Male/ Female	Total
Inborn errors of metabolism	504	1404	42	42	1270/722	1992
Neuromuscular and neurodegenerative disorder	87	395	47	356	627/258	885
Haematological	7	121	2	19	96/53	149
Pulmonary	44	28	1	2	43/32	75
Musculoskeletal	18	23	0	2	25/18	43
Dermatological	6	12	2	10	13/17	30
Congenital hearing loss	0	12	0	17	10/19	29
Ophthalmic	0	4	0	16	9/11	20
Endocrine	4	14	0	2	10/10	20
Hepatic	6	6	0	3	12/3	15
Nephrological	1	0	3	11	11/4	15
Mitochondrial	0	5	2	5	9/3	12
Cardiological	2	0	0	3	3/2	5
Immunological	0	4	0	0	3/1	4
**Total**	**679**	**2028**	**99**	**488**	**2141/1153**	**3294**

### Technological impact on diagnostic yield

Overall, 305 genetic diseases in 3294 patients were identified in the present study cohort from years 2000 to 2022. The disorders were classified into 14 groups based on the major organ system or organ affected (Table 2). These included neuromuscular and neurodevelopmental disorder (NMND), inborn errors of metabolism (IEM), haematological, pulmonary, musculoskeletal, dermatological, congenital hearing loss, ophthalmic, endocrine, hepatic, nephrological, mitochondrial, cardiological and immunological. Majority of the genetic disorders identified in the present study were under the NMND group (D = 149), followed by the IEM group with 47 disorders. Majority of the disorders identified in the study were autosomal recessive (D = 180, 59.2%) followed by autosomal dominant (D = 107, 35.1%,). X-linked disorders constituted 5.7% of the total cases (D = 16) and two disorders showed mitochondrial mode of inheritance (Additional file 4 and 5).

**Table 2:** Stratification of diagnosed patients by disease groups across 22 year period

	2000	2001	2002	2003	2004	2005	2006	2007	2008	2009	2010	2011	2012	2013	2014	2015	2016	2017	2018	2019	2020	2021	2022	Total cases
Inborn errors of metabolism	3	10	10	19	9	15	13	39	39	38	60	71	81	98	124	184	186	176	188	176	90	176	187	1992
NMND									19	24	41	26	51	69	61	63	61	71	38	82	63	86	130	885
Haematological										8	16	20	22	22	16	9	8	7	3	4	4	7	3	149
Pulmonary									5	2	9	3	4	4	12	6	7	1		1	4	8	9	75
Musculoskeletal												1	2	1		2	4	2	8	2	2	8	11	43
Dermatological													1	1	2	2	1	2	1	5	4	1	10	30
Congenital deafness															1		1		1	3	3	10	10	29
Ophthalmic																1			1	5	2	6	5	20
Endocrine														1		1	2	2	2	2	4	4	2	20
Hepatic														1		1	1	2	2	1	2	2	3	15
Nephrological																	2		2	3	3	3	2	15
Mitochondrial															1	3	1		1	1	1	2	2	12
Cardiological																		1		1			3	5
Immunological																1						2	1	4
**Total**	3	10	10	19	9	15	13	39	63	72	126	121	161	197	217	272	274	263	247	285	182	313	374	3294
**Total cases referred**	10	27	24	37	28	41	45	145	192	180	303	341	367	403	546	592	583	635	618	662	415	517	770	7481
**Diagnostic yield (%)**	30.0	37.0	41.7	51.4	32.1	36.6	28.9	26.9	32.8	40.0	41.6	35.5	43.9	48.9	39.7	45.9	47.0	41.4	40.0	43.1	43.9	60.5	48.6	

It is noteworthy that before year 2015, the proportion of patients were mainly identified under the IEM, NMND and haematological group (Table 2; Additional file 6 and 7). It is only after the introduction of CES and WES based tests in 2015 that a significant number of patients with rare genetic diseases were identified under the NMND, congenital hearing loss, dermatological, hepatic and nephrological disorder groups. Additionally, introduction of the NGS technology impacted the diagnostic yield as we observed an increase from 30% in 2000 to 49.1% in 2022 (Table 2; Additional file 6 and 7) with an average diagnostic rate of 40.8% was achieved in the present study cohort throughout the 22-year period. A total of 186 cases and 134 cases were diagnosed using CES and WES, respectively, in the present study. Critically, whilst NMND group constituted on an average 60.5% of all cases diagnosed across 14 disease groups each year using CES/WES between 2015 and 2022, the proportion of all NMND cases diagnosed with these technologies increased from 15.9% in 2015 to 54.6% in 2022. This suggests a significant rise in detection of rare neurological and neurodevelopmental disorders that were missed previously by conventional approaches (Additional file 5–8).

### Genetic disorder prevalence by disease groups

**Fig. 2:**
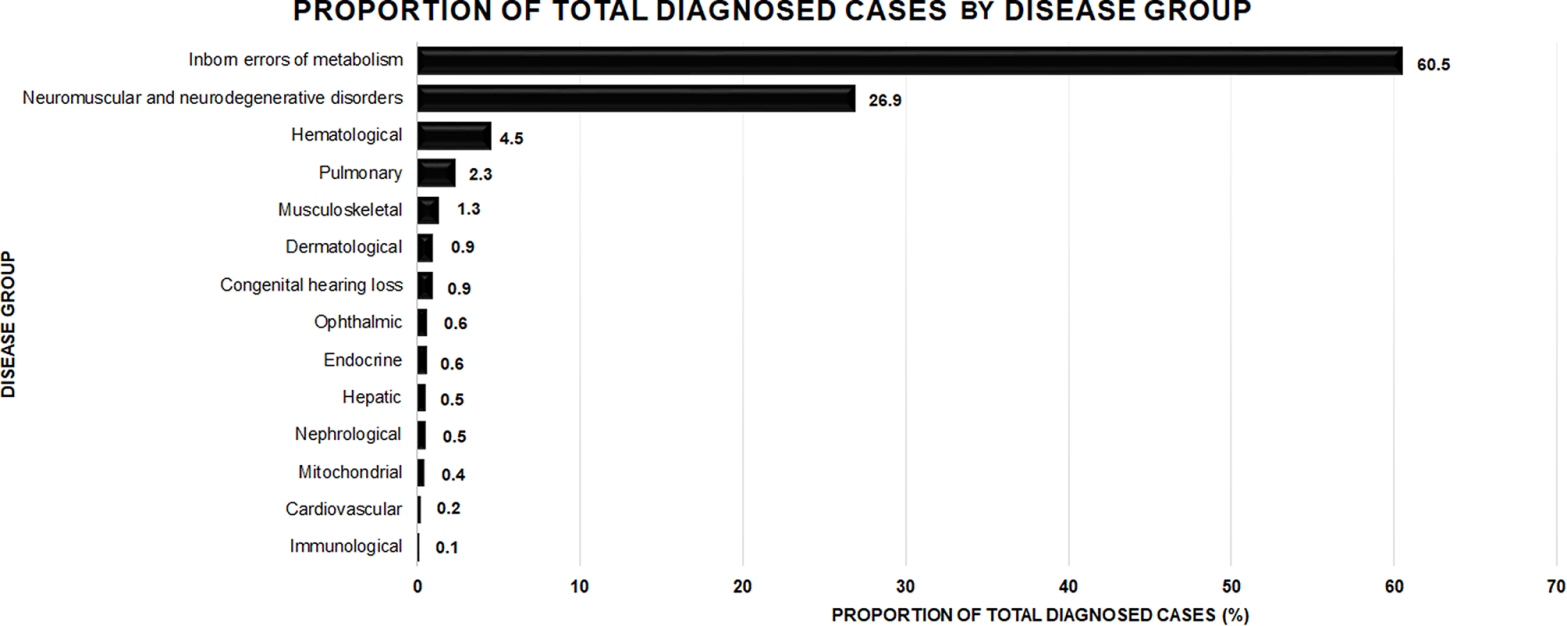
Percentage distribution of patients with rare genetic diseases diagnosed under the 14 disease groups

In the present cohort, we observed highest number of cases diagnosed under the IEM (*N* = 1992; 60.5%) and NMND (*N* = 885; 26.9%) groups. Figure 2 gives the percentage distribution of patients under the 14 disease groups. Interestingly, majority of the patients diagnosed under the NMND group were referred from Gujarat whereas, patients diagnosed under the IEM group were referred from across India (Additional file 3) as our centre represents one of the major referral centres for lysosomal storage disorders across the country. Excluding the NMND and IEM group, 13% of the total diagnosed cases were distributed across the remaining 12 disease groups. Major centres that referred these patients were from Gujarat followed by Maharashtra and Rajasthan (Additional file 3).

The number of patients diagnosed with DMD (OMIM#310200; *N* = 291/885; 32.9%) and SMA (*N* = 141/885; 15.9%) were the highest under the NMND group. Notably, 242 patients (27%) were diagnosed with one of the trinucleotide repeat expansion disorders, the highest of which was spinocerebellar ataxia (SCA) (*N* = 85/885; 9.6%) (Table 3). SCA2 cases (OMIM#183090) were the most common amongst SCA types. Less common muscular dystrophies identified in this cohort included limb-girdle muscular dystrophy (LGMD) (*N* = 23/885, 2.6%) with causative variants identified in 6 different genes (Additional file 4). Spastic paraplegia 11 (OMIM#604360) was the most common (*N* = 6/12; 50%) among hereditary spastic paraplegia in the present study. In total, 82 patients were diagnosed with neurodevelopmental disorders (Additional file 4). Among IEM disorders, majority of the patients were diagnosed with Gaucher disease (*N* = 224/1992, 12%) followed by other LSDs (Table 3) and small molecule IEM disorders (Additional file 5).

**Table 3:** Common genetic variants identified across rare genetic disorders

Disorder group	Disease name	OMIM	Number of patients	Gene	Common mutation identified	Percentage of cases
Neuromuscular and neurodevelopmental disorders	Duchenne muscular dystrophy	#310200	291	*DMD*	Exon 45 to exon 52 deletion	58.2% (*N* = 169/291)
	Spinal muscular atrophy	#253300	141	*SMN1*	Exon 7 and exon 8 deletion	100% (*N* = 141/141)
	Myoyonic dystrophy type I	#160900	82	*DMPK*	NA	NA
	Spinocerebellar ataxia 2	#183090	38	*ATXN2*	NA	NA
	Spinocerebellar ataxia 3	#109150	29	*ATXN3*	NA	NA
	Spinocerebellar ataxia 1	#164400	10	*ATXN1*	NA	NA
	Huntington’s disease	#143100	37	*HTT*	NA	NA
	Friedrich ataxia	#229300	19	*FXN*	NA	NA
	Fragile-X	#300624	19	*FMR1*	NA	NA
	Limb girdle muscular dystrophy type 1	#253600	8	*CAPN3*	No common variant identified	NA
	GNE myopathy	#605820	9	*GNE*	c.2179G > A (p.Val727Met)	100% (*N* = 9/9) *Heterozygous
	Spastic paraplegia 11	#604360	6	*SPG11*	c.267G > A (p.Trp89Ter)	50% (*N* = 3/6)
Inborn errors of metabolism	Gaucher disease	#230800	224	*GBA*	c.1448T > C (p.Leu483Pro)	60% (*N* = 134/223)
	MPS IVA	#253000	209	*GALNS*	c.230C > G (p.Pro77Arg)	56% (*N* = 14/25)
	Niemann-Pick disease A/B	#257200/ #607616	167	*SMPD1*	No common variant identified	NA
	GM1 gangliosidosis	#230500	141	*GM1*	No common variant identified	NA
	MPS II	#309900	133	*IDS*	No common variant identified	NA
	Metachromatic leukodystrophy	#250100	136	*ARSA*	No common variant identified	NA
	Tay-Sachs disease	#272800	126	*HEXA*	c.1385A > T (p.Glu462Val )	22% (*N* = 11/48)
		#272800		*HEXA*	c.1278insTATC	16% (*N* = 8/48)
		#272800		*HEXA*	c.964G > T (p.Asp322Tyr)	6% (*N* = 3/48)
	Mucolipidosis II/III	#252500/ #252600	79	*GNPTAB*	c.3503_3504delTC (p.Leu1168GlnfsTer5)	34% (*N* = 12/35)
Hematologic disorders	Beta-thalassemia	#613985	120	*HBB*	c.92 + 5G > C, c.316 − 149_*342delinsAAGTAGA, c.126_129del, c.27dupG, c.92 + 1G > T	85% (*N* = 102/120)
	Hemophilia A	#306700	16	*F8*	Intron 22 inversion	42% (*N* = 5/12)
Pulmonary disorders	Cystic fibrosis	#219700	74	*CFTR*	c.1521_1523delCTT (deltaF508)	66% (*N* = 49/74)
Musculoskeletal disorders	Achondroplasia	#100800	21	*FGFR3*	c.1138G > A	71% (*N* = 15/21)
				*FGFR3*	c.1138G > C	29% (*N* = 6/21)
	Osteogenesis imperfecta type I/II/III/IV	#166200, #166210, #259420, #166220	1	*COL1A1*	No common variant identified	NA
	Osteogenesis imperfecta type I/II/III/IV	#166200, #166210, #259420, #166220	2	*COL1A2*	No common variant identified	NA
	Osteogenesis imperfecta type V	#610967	2	*IFITM5*	No common variant identified	NA
	Progressive pseudorheumatoid dysplasia	#208230	3	*CCN6*	c.298T > A (p.Cys100Ser)	100% (*N* = 3/3)
Dermatological disorders	Oculocutaneous albinism type IA/ IB	#203100/ #606952	20	*TYR*	c.832C > T (p.Arg278Ter)	30% (*N* = 6/20)
	Congenital ichthyosis-1	#242300	4	*TGM1*	No common variant identified	
Congenital hearing loss	Deafness, autosomal recessive	#220290	8	*GJB2*	c.71G > A (p.Trp24Ter)	37% (*N* = 3/8)
	Usher syndrome type IIA	#276901	4	*USH2A*	NA	NA
Ophthalmic disorders	Retinitis pigmentosa-54	#613428	3	*PCARE*	No common variant identified	NA
Endocrinological disorders	Congenital adrenal hyperplasia	#201910	16	*CYP21A2*	c.29–13C > G	43% (*N* = 7/16)
Hepatic disorders	Gilbert syndrome	#143500	13	*UGT1A1*	A [TA]7 TAA	46.2% (*N* = 6/13)
	Crigler-Najjar syndrome, type I	#218800				
Nephrological disorders	Alport syndrome	#301050	5	*COL4A5*	c.2918-11G > A	40% (*N* = 2/5)
Mitochondrial disorders	Leber’s Hereditary Optic Neuropathy	#535000	3	*MTND4*	G11778A	100% (*N* = 3/3)
	Leigh syndrome	#500017	3	*MTATP6*	No common variant identified	NA

We observed majority of the patients diagnosed with β-thalassemia (OMIM#613985; *N* = 120/149; 80.5%), cystic fibrosis (OMIM#219700; *N* = 74/75; 98.7%) and achondroplasia (OMIM#100800; *N* = 21/43; 48.8%) under the haematological, pulmonary, and musculoskeletal disease groups, respectively (Table 3). Likewise, oculocutaneous albinism type IA (OMIM#203100; *N* = 6/30; 20%), congenital hearing loss due to biallelic *GJB2* gene variant (OMIM#220290; *N* = 8/29; 27.6%), retinitis pigmentosa (*N* = 5/20; 25%) and congenital adrenal hyperplasia (CAH) (OMIM#201910; *N* = 16/20; 80%) represented the highest cases under the dermatological, congenital deafness, ophthalmic and endocrine groups, respectively. Gilbert syndrome (OMIM#143500; *N* = 6/15; 40%) and Alport syndrome (*N* = 5/15; 33.3%) were common disorders identified under the hepatic and nephrological groups, respectively (Table 3).

### Variant distribution amongst genetic disorders

Assessment of the genotype and variant details of the patients diagnosed with rare genetic disorders revealed commonly occurring and founder variants for particular disorders. In 58.2% (*N* = 169) of the patients diagnosed with DMD disease, hemizygous deletion encompassing exons 45 to exon 52 of the *DMD* gene was observed. Another common variant c.1448T > C (p.L483P) in the *GBA* gene was detected in approximately 60% (*N* = 134) of all patients diagnosed with Gaucher disease. Table 3 summarises information on the common variants detected in corresponding genes in patients diagnosed with rare disorders under the 14 disease groups. In addition to this, 82 distinct variants in 63 genes were identified in patients with neurodevelopmental disorders (Additional file 4). Variant and genotype details of patients diagnosed under haematological, pulmonary, musculoskeletal, dermatological, congenital hearing loss, ophthalmic, endocrine, hepatic, nephrological, mitochondrial, cardiological and immunological groups have been described in Additional file 5.

## Discussion

In recent years, increased use of DNA sequencing in clinical settings has resulted in identifying the genetic basis of several rare genetic diseases [2, 56]. Improvement in technology has contributed towards development of high throughput sequencing technologies, at an affordable cost [57]. Coupled with this, several therapeutic options are now available for some of these rare diseases (Table 4). Currently, there are 35 major clinical research institutions in India, including our institute, which are working on different rare disease groups [17]. Commonly addressed among these are dermatological disorders, mitochondrial disorders, neurological, neuromuscular, LSDs, IEMs, haematological disorders, musculoskeletal and ophthalmic disorders. The aim of the present study was to understand the burden and genetic epidemiology of these rare genetic diseases diagnosed at a tertiary genetic centre, in India over a period of 22 years.

**Table 4:** Therapeutic modalities currently available for rare genetic diseases

Treatment modality	Principle	Conditions for which there is clinical approval	Reference
Substrate reduction therapy	Inhibiting the biosynthesis of Storage metabolites in the lysosomes by using small molecules.	Gaucher disease, Fabry disease, Niemann-Pick type C, MPS-IIIB	[124]
Enzyme replacement therapy	Recombinant enzymes that are modified to provide a longer half-life, more potent activity, resistance to degradation or targeting to a specific organ, tissue or cell type	Gaucher disease, Fabry disease, MPS I, MPS II, Pompe disease, MPS VI, Wolman disease, Batten disease, MPS IVA, MPS VII and α-mannosidosis	[125]
Oligonucleotide therapies	Targeting RNA to reduce the production of a specific disease-associated protein by promoting degradation of its mRNA.	SMA and DMD (Nusinersen, Eteplirsen)	[126]
Gene therapy	A vector is used to express a transgene (with the endogenous sequence or codon optimized) that encodes the desired protein, under the control of an appropriate promoter	SMA, hemophilia A, hemophilia B, adrenoleukodystrophy, β-thalassemia, sickle cell disease	[127]
Hematopoietic stem cell therapy	The ability of the transplanted cells and/or their progeny to contribute to fixed-tissue macrophage populations in the affected tissues and to become local permanent sources of functional lysosomal enzymes.	MPS I, MPS II, metachromatic leukodystrophy, Krabbe, β-thalassemia, sickle cell disease, Gaucher disease, MPS IVA, epidermolysis bullosa	[128]
Iron chelation therapy	To maintain safe levels of body iron at all times, by balancing iron intake from blood transfusion with iron excretion by chelation	β-thalassemia	[129]

### Neuromuscular and neurological disease group

A high burden of DMD and SMA cases that were documented in the present cohort was also observed previously in a neuromuscular cohort study in Lebanon, demonstrating SMA in 40.3%, followed by DMD in 17% of its patients [10]. Of note, a recent study by Nilay et al. 2020 showed carrier frequency of 1 in 38 for SMA in the North Indian population [20], higher than that previously reported in the US, Australia, Europe and UK [58, 59]. This is probably one of the reasons for the high SMA cases in the country and in the present cohort. Interestingly, the mutation spectrum seen for DMD in the present cohort is similar to that described previously in the DMD global database, with exon 45 deletion identified in 68% cases, being the most common [60]. Likewise, exon 7–8 deletion of the *SMN1* gene was reported in 90.7% of the total SMA cases in the NMD cohort in Lebanon [10], which is in concordance with our present observations and earlier study [61].

Of note, we observed *CAPN3* and *DYSF* as recurrently mutated genes among the LGMD patients in our cohort. This is in congruence with the observations of Nallamilli et al. whereby 17% and 16% of the 4656 LGMD patients presented with variant(s) in the *CAPN3* and *DYSF* genes, respectively [62]. Importantly, the founder variant c.2338G > C in the *CAPN3* gene, previously reported in the Indian Agarwal community [63], was seen in only two patients in a compound heterozygous state in the present cohort, suggesting that the mutation spectrum for *CAPN3* gene is likely to be distinct among different sub-populations in the country. Contrary to this, variant c.2179G > A (p.Val727Met) in the *GNE* gene was observed in all patients with GNE myopathy (OMIM#605820) in the present study in compound heterozygous state. This variant has previously been reported to be a common variant amongst Indian patients of GNE myopathy [64]. This adds further evidence of probability of a founder effect for GNE myopathy in India, although haplotype analysis is beyond the scope of the current study.

### Trinucleotide repeat expansion disorders

Of the NMND disease group, trinucleotide repeats expansion disorders were identified in 27% cases (*N* = 242). A significantly high proportion of SCA cases, particularly SCA2 (*N* = 37) in the present cohort is likely to be due to the founder effect [65]. Similarly, whilst the SCA12 founder mutation has been reported in the Indian Agarwal community [66], a significantly low numbers of SCA12 cases were observed in the present study. This could be due to limited number of referrals from the eastern geographical region of the country, where the Agarwal community predominantly resides. Following the study of Bhowmik et al. 2016, we present here the largest series of patients (*N* = 82) with myotonic dystrophy type I from India [67]. Likewise, we observed a significant number of Huntington disease cases in the present cohort. This observation is supported by another study where Chheda et al. 2018 showed a high prevalence of 49% in 503 pan-India patients suspected with Huntington disease [68]. The average range of CAG repeats on the expanded allele of the *HTT* gene observed in the Indian patients with Huntington’s disease is 41–59 [69], which was also observed in present cohort.

### Neurodevelopmental disorders

Strikingly high proportion of neurodevelopmental disorder (NDD) cases were reported in a recent population based study across five regions in India [70]. The high proportion of NDD cases observed in the present cohort further supports prior observation. Furthermore, we observe an increase in detection of NDD due to the use of NGS based CES/ WES approaches, with causative variants in genes associated with autosomal dominant or X-linked phenotypes [71, 72]. Primary mode of inheritance for these rare NDDs is *de novo* with the variant reported to be occurring on the paternal allele in majority of the cases [73] However, it is beyond the scope of the present study to record the paternal age at proband’s conception. Interestingly, the genes in which causative variants were identified in present study encode transcriptional and chromatin regulators, translation initiator factors, ion channels, synaptic proteins and neuronal migration machinery, which is in congruence with the reported literature [71, 74].

### Inborn errors of metabolism group

The distribution of different LSDs in the present study is similar to that previously reported by our and other groups in the country [75, 76], with Gaucher disease being the most common LSD followed by the MPS disease group. Also, common variants identified in present study cohort for LSDs like Gaucher disease, MPS I, MPS II and Mucolipidosis II/III is in concordance with data reported by other groups in India [77–79]. Critically, several variants for Niemann-Pick A/B, GM1 gangliosidosis, Krabbe, Tay-Sachs and MPS IVA were enriched in certain sub-populations, with two variants being shown to be founder variants from 2 communities in Gujarat [80, 81].

Pilot studies for newborn screening across India detected high prevalence of small molecule IEMs such as glucose-6-phosphate dehydrogenase (G6PD) deficiency, amino acid disorders and organic acidemias [82–84]. However, low proportion of patients with this disease were detected on the current study which is likely to be due to exclusion of cases diagnosed with tandem mass spectrometry and/or gas chromatography mass spectrophotometry only.

### Haematological group

India contributes significantly to the global burden for β-thalassemia with estimates suggesting that approximately 32,400 children each year are born with hemoglobinopathies. Interestingly however, we observed a higher proportion of β-thalassemia carriers (75%) compared to affected cases (13%) in the present cohort. Indeed, the proportion of overall cases stratified in the haematological group increased from 0% in 2000–2004, peaked at 11.7% in 2010–2014 and reduced to 1.6% in 2020–2022 (Additional file 6). This inverted U shaped curve follows the national and state government initiatives on increasing disease awareness of thalassemia which led to the paradigm shift from proband diagnosis to parental screening and prenatal diagnosis. Additionally, the multicentre Jai Vigyan programme established by the Indian Council of Medical Research helped to strengthen screening testing centres in different states for community control of thalassemia [85]. Moreover, the number of centres offering testing services for β-thalassemia has increased over the past decade, which is also a probable reason for the poor representation of these cases at our centre. Nonetheless, the mutation spectrum observed in these patients was similar to that reported by other groups in the country, with c.92 + 5G > C being the most common *HBB* gene variant [86].

### Pulmonary group

Population prevalence estimates of 1 in 43,321 to 1 in 100,323 have been carried out previously for cystic fibrosis (CF) [87]. The present cohort had 98% (*N* = 74) of patients diagnosed with CF under the pulmonary group. Of all the variants reported in the *CFTR* gene, 66% of the patients with CF were detected with the *CFTR*:c.1521_1523del variant, which has an estimated frequency of 19–34% in the Indian CF patients [88, 89]. This observation suggests that patients suspected with CF could be tested for *CFTR*:c.1521_1523del variant only in the beginning, due to the high probability of detection, with a subsequent reflex testing for the entire gene if the variant is not detected in the patient.

### Musculoskeletal group

A high proportion of patients in the musculoskeletal group with a diagnosis of achondroplasia or osteogenesis imperfecta in the present cohort is in congruence with prior observations in Indian patients [90]. Of note, three unrelated patients but belonging to the same ethnic community- Gujarati Patni- were diagnosed with progressive pseudo rheumatoid dysplasia (PPD; OMIM#208230) due to the presence of the same variant c.298T > A in the *CCN6* gene [91]. However, the aforementioned variant was not detected in a large series of 79 patients with PPD from southern India, which primarily consisted of two common variants c.233G > A and c.1010G > A in the *CCN6* gene [92]. This suggests possible founder variants in ethnic communities residing in two distinct geographical locations, however, the assessment of the same is beyond the scope of the current study.

### Dermatological group

Observation of oculocutaneous albinism type 1 A (OMIM#203100) being commonly observed dermatological group disease in the current study is in congruence with the observation by another study from India [93]. Pathogenic variants in the *TYR* gene were detected in 84.7% of the total cases in their cohort, which is similar to observation in present study. A founder variant c.832C > T in the *TYR* gene, reported in the Tilli population in eastern India was also observed at a high frequency (27%) in the present study cohort. Surprisingly, this variant has been reported in several other populations [94, 95] indicating that this is a recurrently occurring variant across multiple ethnic populations. Although, it is possible that this variant has also accumulated as a regional founder mutation because of its high allele frequency in the gnomAD database for the South Asian population as compared to African and European populations.

### Congenital hearing loss group

More than 125 genes are associated with non-syndromic hearing loss, of which 70 genes have been associated with autosomal recessive non-syndromic hearing loss (AR-NSHL). *GJB2*-related AR-NSHL is the most common genetic aetiology in Asian and European population [96]. This is also evident by the highest percentage of *GJB2* gene variants identified in the present study cohort. Furthermore, a probable founder variant c.71G > A in the *GJB2* gene [97] was detected in 26% of the patients in our cohort. This variant is also a common variant in the Pakistani population as well as in the Roma population of Slovakia probably because of close correlation to the Indian origin of these populations [98].

### Ophthalmic group

Retinitis pigmentosa (RP) has been reported as the most common ophthalmic group disorder in Indian population previously [99]. A comparative study to assess the prevalence of inherited retinal dystrophies (IRDs) in patients from India and USA [100] showed RP, Leber congenital amaurosis (LCA) and Stargardt’s disease to be the most common IRDs. Whilst patients within ophthalmic group represented a small proportion of the overall number of patients across the 14 disease groups, majority of the patients within ophthalmic group were diagnosed with RP and LCA. Critically, a significant proportion of patients with RP had variants in the *PCARE* and *RPE65* genes, which is in congruence with prior literature observation [100].

### Endocrine disorder group

Congenital adrenal hyperplasia due to 21-hydroxylase deficiency (CAH; OMIM#201910) represented the largest proportion of patients within endocrine disorder group, similar to the prior report from India [82]. Of note, regional variation in the CAH prevalence has been noted in India and prior genetic study in these patients presented c.293–13 C > G variant in the *CYP21A2* gene as a common variant; similar observation was made in our cohort too [101, 102]. This presents an opportunity to assess the common variant on the outset followed by *CYP21A2* gene sequencing as reflex test, if required.

### Hepatic disease group

Gilbert syndrome (OMIM#143500) has been reported to be the most common hereditary unconjugated hyperbilirubinemia due to a common promoter variant (TA)7/7 of the *UGT1A1* gene [103]. Within the hepatic disease group of the current study, 46% (*N* = 6/13) of the patients presented with the aforementioned variant during genetic test. Critically, stronger association between the presence of homozygous (TA)7/7 allele in the *UGT1A1* gene and risk of Gilbert syndrome has been observed in Indian population compared to other ethnic groups [104, 105]. This in part could explain the high incidence of Gilbert syndrome within the current cohort.

### Nephrological group

Inherited kidney diseases account for ~ 10–15% of adult patients undergoing kidney replacement therapy [106, 107]. In present cohort, among patients with nephrological disorders, 33% of cases were diagnosed with Alport syndrome followed by polycystic kidney disease (PKD). Previously, Alport syndrome and PKD have been shown to be a common cause of hereditary nephropathy in an Indian cohort of patients with chronic renal failure as a key phenotypic feature [96]. Additionally, Alport syndrome was also one of the common genetic diagnoses (14%) in a cohort of 76 Indian children suspected with kidney disorders [108]. This suggests an overall high prevalence of Alport syndrome in patients with chronic kidney diseases in India.

### Mitochondrial disease group

An interesting observation within this group was that majority of the adult onset disease patients presented with variants in the mtDNA genes compared to nuclear DNA genes, which is in concordance with prior literature observation [109]. Critically, several recent studies have shown a high diagnostic yield in patients suspected with mitochondrial disease using WES [110]. However, not all patients suspected of mitochondrial disease were subjected to WES, which in turn could likely explain the low diagnostic yield within the mitochondrial disease group.

### Cardiological group

Inherited cardiac disorders, broadly classified into cardiomyopathies and channelopathies have a prevalence of 3% worldwide [111]. The American Heart Association guidelines for the diagnosis and treatment of hypertrophic cardiomyopathy (HCM) in 2011 recommended genetic testing for the management of HCM as ~ 40% of the cardiomyopathies are likely to be genetic in origin [112]. Literature evidence suggests that a causative variant is identified in genes encoding a sarcomeric protein in up to 60% of individuals with HCM, with *MYBPC3* or *MYH7* being the most common genes [113]. Likewise, available data from India showed that variants in the *MYH7*, *TNNT2* and *MYBPC3* genes accounted for one third of the total reported variants [114]. Currently however, clinical application of genetic testing in cardiology practice especially in cases with sudden cardiac death is at a nascent stage in comparison to that in paediatric clinics for metabolic and other neurological conditions. This likely to be one of the reasons for the poor presentation of this group in present cohort. Despite our small cohort of 5 patients, we could also identify one patient with a pathogenic variant in the *MYBPC3* gene. Thus, increase in uptake of genetic testing is necessary to delineate the genetic architecture for Indian patients with cardiomyopathy.

### Immunological disease group

The present cohort had a very poor representation of inherited immunological disorders. One contributing factor can be lack of awareness of inherited immunological disorders, especially among general practitioners, paediatricians and adult specialists. The second critical reason could be high mortality rate in these patients [115] before a diagnosis is achieved. Hence, there is a dearth of referrals for genetic testing in patients presenting with recurrent and severe infections, as most of them are likely misdiagnosed and treated as cases of infectious diseases [116]. Recent review from India suggests X-linked Agammaglobulinemia, severe combined immunodeficiency and Wiskott-Aldrich syndrome to be commonly observed immunodeficiency disorders [116]. With increase in awareness and subsequent update in genetic testing there will likely be improved epidemiological estimates of these diseases in India.

### Future perspectives

Several countries have used different approaches such as administrative hospitalization data or cohort-based study statistics to estimate the prevalence of rare diseases [117]. The present retrospective data here provides information on the distribution of different genetic conditions diagnosed at a tertiary genetic clinic over 22 years’ duration. The data shows a high prevalence of monogenic conditions namely DMD, SMA, β-thalassemia and sickle cell anaemia, as well as, LSDs such as Gaucher disease and MPS disorders. Recently, a national rare disease registry established by the ICMR, Government of India has included all of the aforementioned disorders to assess the national burden. The awareness of different rare genetic disorders, its prevalence, the available diagnostic tests and treatment options is important in order to help draft future health policies, including newborn screening programs as well as choice of diseases for screening in premarital/ preconception stages.

Secondly, information on founder and common mutations identified for several diseases within certain ethnic communities in the present study cohort can be used for the development of affordable, targeted and scalable genetic testing pathways, similar to that developed for the Ashkenazi Jewish population [118]. In contrast, application of “hypothesis free” based assays such as DNA microarray and NGS is critical for genetic diagnosis of disorders with a broad genetic aetiology, such as NDD, intellectual disability and autism spectrum disorder [119]. Even targeted gene panels could be critical in improvement of diagnostic yields in diseases with overlapping phenotypes but different genetic aetiologies such as LSDs [120].

In view of upcoming treatment modalities available for several rare diseases, it is imperative to have molecular epidemiology data of these patients. Lastly, prevention by prenatal genetic screening in families with a history of rare genetic diseases has gained importance and molecular epidemiological data could help in providing targeted interventions in certain communities and/or geographical regions with a high burden of genetic disorders.

### Limitations

Despite the unbiased retrospective methodology used in the present study, several limitations are to be noted. First, a high proportion of patients were diagnosed with LSDs, which is likely to be due to referral bias since the institute is one of the national referral centres for LSDs. Despite this, several impactful work on the distribution of LSD burden, diagnostic assay development and therapeutic interventions in India have been carried out by the institute [121]. Second, due to referral bias, referrals for patients suspected with other disease groups were significantly fewer compared to LSDs. Due to this, accurate and reliable estimates of disease burden pertaining to these disease groups would be untenable. Nonetheless, majority of the observations on disease prevalence and molecular epidemiology are in congruence with the prior literature data as described above. Third, patients with chromosomal abnormalities, sporadic cancer and newborns with inborn errors of metabolism diagnosed only with TMS/ GCMS were excluded. Since their aetiology is primarily sporadic or *de novo* in origin, accurate estimation of disease incidence and burden within the population is beyond the scope of the current study. In contrast, a high proportion of patients with an autosomal recessive disorder within the current cohort could be attributed to the practice of endogamy amongst several ethnic groups in India [12, 122, 123]. Fourth, genetic diagnosis of a given disorder is based upon the sensitivity, specificity and availability of certain genetic diagnostic assays that are available at a given time period. The availability application of NGS based assay at the institute post 2015 suggests underestimation of NDD within the current cohort. Nonetheless, the genetic architecture of the NDD disease patients within the current cohort is in congruence with the data from other populations, suggesting a shared genetic aetiology of these diseases.

## Conclusion

The present 22 years retrospective study of patients diagnosed with rare genetic diseases at a tertiary genetic centre in India, showed the distribution of genetic diseases under different disorder groups. We observed high burden of IEM based disorders such as LSDs followed NMND group consisting of DMD and SMA. The data provides valuable insights into plausible avenues of implementation of affordable diagnostic pathways, implementation of NGS based assays in improving the diagnostic yield of NDD, development of treatment modalities for commonly observed variants and deployment of awareness, genetic counselling and disease prevention programs in communities with a high burden of a certain genetic disorder. With the high number of diseases being observed with a common genetic aetiology, efforts could be directed for research in avenues of novel drug development for these disorders. Lastly, collaboration among the clinical, research and diagnostic communities is needed to address the challenges of diagnosis, management, treatment and prevention of rare diseases in the country.

### Electronic supplementary material

Below is the link to the electronic supplementary material.

Supplementary Material 1

Supplementary Material 2

Supplementary Material 3

Supplementary Material 4

Supplementary Material 5

Supplementary Material 6

Supplementary Material 7

## Data Availability

All data supporting the findings of this study are available within the paper and it’s Supplementary Information.

## Abbreviations

IEM: Inborn errors of metabolism
LSDs: Lysosomal storage disorders
NMND: Neuromuscular and neurodevelopmental disorders
CES: Clinical exome sequencing
WES: Whole exome sequencing
TMS: Tandem mass spectrometry
GCMS: Gas chromatography mass spectrometry
