# Supplementary material for: Burden of rare genetic disorders in India: twenty-two years’ experience of a tertiary centre
Source: Orphanet J Rare Dis. 2024 Aug 13;19:295. doi: 10.1186/s13023-024-03300-z (PMC11323464; doi:10.1186/s13023-024-03300-z)
Supplement: Supplementary file 6 — Supplementary Material 6 [file 13023_2024_3300_MOESM6_ESM.docx]

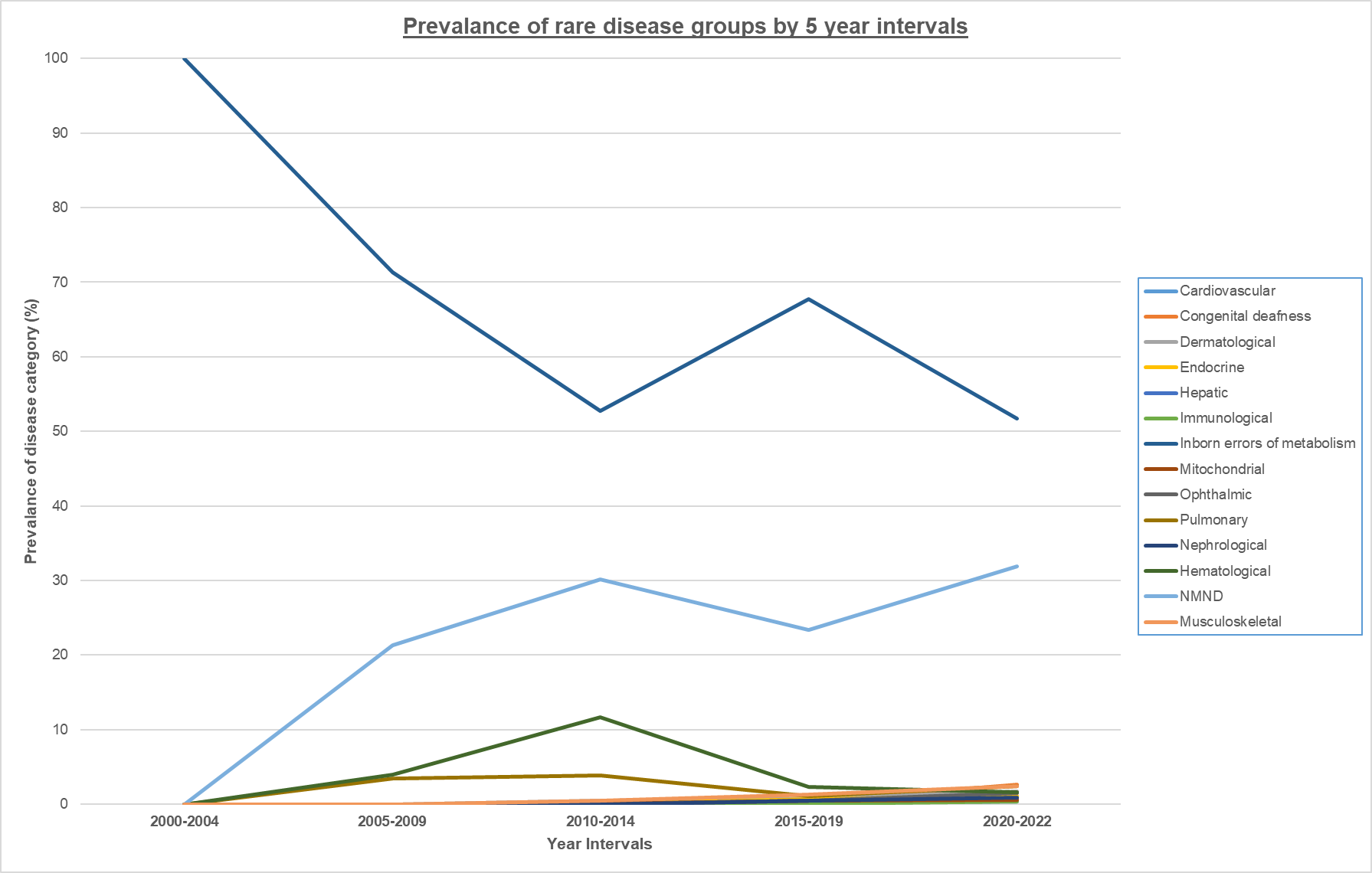


**Figure 1:** Proportion of rare diseases diagnosed in 5-year intervals in the study cohort.


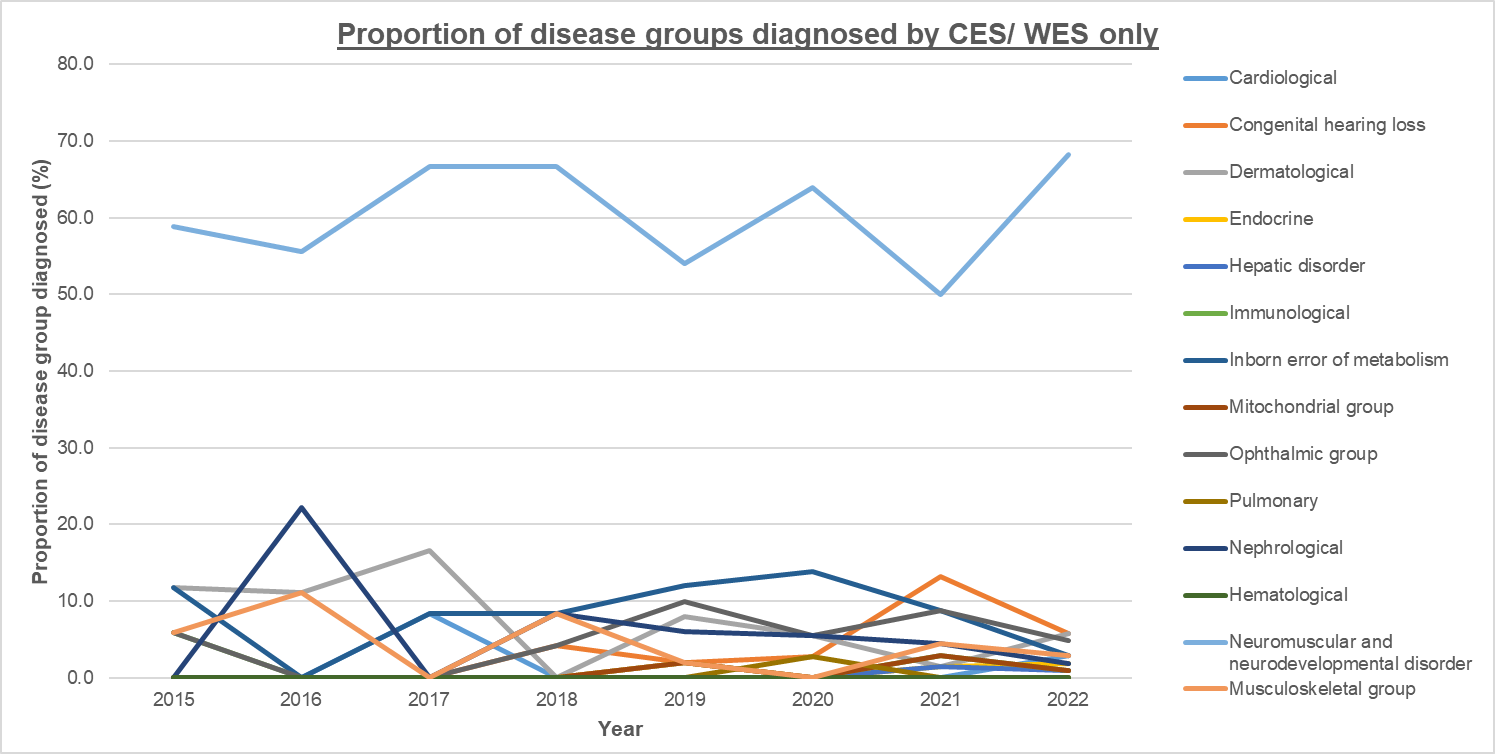


**Figure 2:** Proportion of diagnosis in rare disease group following introduction of clinical exome sequencing or whole exome sequencing.


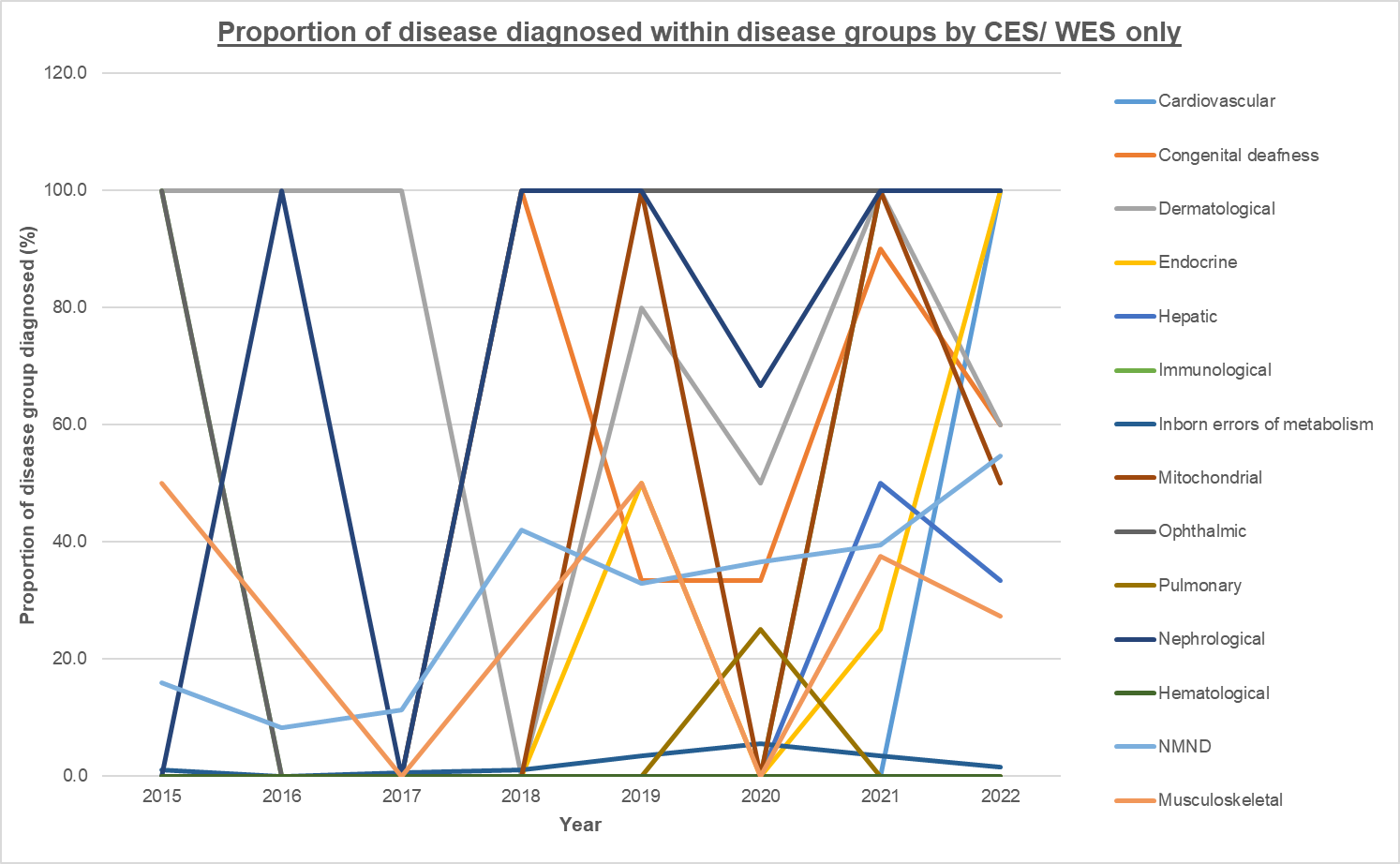


**Figure 3:** Proportion of number of overall diseases diagnosed by clinical exome sequencing or whole exome sequencing within a given disease group.
